# Cataract: Its Nature, Symptoms, &c.

**Published:** 1837-01

**Authors:** 


ART. XII.—*Cataract: a familiar Description of its Nature, Symptoms, and ordinary Modes of Treatment, particularly with reference to the System, &c.* By JOHN STEVENSON, Esq. *Second Edition.—London,* 1836. 12mo. pp. 136.

MOST of our readers are familiar with the name, at least, of JOHN HARRISON CURTIS, ESQUIRE, Aurist, it being as little practicable to escape acquaintance with his name as with that of ROBERT WARREN or DR. MORRISON, or of our admirable friend MECHI, of the magic strop; and for precisely the same reason. Many are, however, probably not aware, as the event, though great, is only of recent date, and the organization of his fame in this department not therefore yet complete,—that this distinguished man, in his progress towards the universal dominion of the senses, has added the province of the eye to his ancient kingdom of the ear. So at least we are informed by the author of the work now before us, who has kindly favoured us, through our publisher, with a copy of his own treatise on one important department of the invaded territory. Mr. Curtis, then, it would appear, has printed a book entitled "On the Physiology and Diseases of the Eye," in which he relates cases, according to the testimony of Mr. Stevenson, attesting the efficacy of a certain "new method of curing cataract without an operation." On this book Mr. Stevenson, as was natural, pounces with vast fury, and tears it all to shreds. The "new method" consists in the application to the eye of a solution of potash, (the strength is not stated, of course,) with which he touches the centre of the cataract from time to time till it disappears.

Mr. Stevenson declares all this to be highly apocryphal and unorthodox, and forthwith proceeds kindly to instruct us respecting "the method devised and carried into extensive operation" by himself, "with almost invariable success." What this method precisely is, is as much a secret as the strength of his rival's solution, and the concealment is just of as much importance in the one case as it is in the other. It would seem, however, that Mr. Stevenson touches the cataract with a needle, while Mr. Curtis touches it with a solution of caustic potash. Stevenson obtains (see page 92,) the patient's confidence, avails himself of a favorable moment, when the patient is not aware of his intention, introduces his instrument into the eye, and completes the operation without exciting a consciousness of what is going on! Not the smallest irritation ensuing, the following day the pupil assumes its natural character, every particle of the cataract having been absorbed during the intermediate night!!

Now this is a feat that even Mr. Curtis, we imagine, cannot outdo, if he can match it. It is really almost, if not altogether, as clever as touching the centre of the cataract "by means of a camel's-hair pencil," which Mr. Curtis has often done, and with an effect "unexpectedly irresistible."

We trust the time is not far distant, if the very publication of such books as we have referred to does not show that it is already arrived, when the diseases of the eye and of the ear shall be either entirely committed to the charge of the scientific and well-educated general surgeon, or, at least, that, if still confided to a class of special practitioners, these shall be men neither ignorant of the principles of medical science, nor disregardful of those which regulate the conduct of honorable men.

